# Health Issues and Immunological Assessment Related to Wuhan's COVID-19 Survivors: A Multicenter Follow-Up Study

**DOI:** 10.3389/fmed.2021.617689

**Published:** 2021-05-07

**Authors:** Qi Mei, Fei Wang, Yang Yang, Guangyuan Hu, Suihuai Guo, Qing Zhang, Amy Bryant, Lingjie Zhang, Christian Kurts, Li Wei, Xianglin Yuan, Jian Li

**Affiliations:** ^1^Department of Oncology, Tongji Hospital, Huazhong University of Science and Technology, Wuhan, China; ^2^Department of Chinese Medicine, Wuhan No.1 Hospital, Huazhong University of Science and Technology, Wuhan, China; ^3^Department of Ophthalmology, Renmin Hospital of Wuhan University, Wuhan, China; ^4^Department of Intensive Care Unit, Wuhan Wuchang Hospital, Wuhan, China; ^5^Covid-19 Rehabilitation Center, Hubei Provincial Hospital of Traditional Chinese Medicine, Wuhan, China; ^6^Department of Biomedical and Pharmaceutical Sciences, College of Pharmacy, Idaho State University, Meridian, ID, United States; ^7^Department of Anesthesiology, Hubei Provincial Hospital of Integrated Chinese and Western Medicine, Wuhan, China; ^8^Institute of Experimental Immunology, University Clinic of Rheinische Friedrich-Wilhelms-University, Bonn, Germany

**Keywords:** post-COVID-19, SARS-CoV-2, mortality, hospital discharge, post-COVID-19 sequela, physical and psychological symptoms, antibody test, IgG and IgM

## Abstract

**Background:** Currently, a large number of hospitalized coronavirus infectious disease-2019 (COVID-19) patients have met the clinical discharge criteria and have been discharged. Little is known about the sequelae and herd immunity, two important factors influencing the life quality and safety of COVID-19 survivors.

**Methods:** Discharged COVID-19 patients from four medical facilities in Wuhan, China, were followed in order to record and investigate possible post-COVID-19 sequelae and herd immunity. After hospital discharge, patients reported to Fangcang shelter hospitals for an initial 14-day period of mandatory clinical monitoring. After release from these shelter hospitals, patients returned home for self-quarantine. Real-time quantitative PCR (RT-qPCR) was used for severe acute respiratory syndrome-related coronavirus 2 (SARS-CoV-2) detection. Colloidal gold-based immunochromatographic strip assay (ICGSA) was used for anti-SARS-CoV-2 immunoglobulin G (IgG) and immunoglobulin M (IgM) antibody testing. The data for this study are derived from case reports, medical records, and self-reports.

**Results:** A total of 3,677 COVID-19 survivors [median age = 59 years, interquartile range (IQR) = 47–68, range = 10–98; 55.5% female] who were released from four hospitals in Wuhan, China, between January 18 and March 29, 2020 were followed for a median of 144 days (IQR = 135–157). During follow-up, 976 (26.5%) patients had at least one post-COVID-19 sequela. The incidence of post-COVID-19 sequelae among elderly COVID-19 survivors (age ≥60 years) was slightly increased compared to that of young COVID-19 survivors (age <60 years; relative risk = 1.05, 95% CI = 1.02–1.10, *p* = 0.007). During follow-up, a dramatic reduction of anti-SARS-CoV-2 IgG (88.0%, 95% CI = 84.2–90.4) and IgM (93.2%, 95% CI = 88.5–96.4) antibodies was observed. Among these COVID-19 survivors, 1.2% (*n* = 45) retested positive for SARS-CoV-2 and 1.0% (*n* = 37) died during follow-up. Of those who died during follow-up, 70.3% were male and all were negative for both IgG and IgM, except for one person who was IgG-positive.

**Conclusions:** Our study documents significant post-COVID-19 sequelae that impair functions of multiple organ systems in COVID-19 survivors, suggesting that the long-term effects of this disease will negatively impact survivors' quality of life, continue to strain health care systems, and result in extended periods of lost productivity. Furthermore, female gender and anti-SARS-CoV-2 immunity may play an essential role in the survival after COVID-19 infection.

## Introduction

The coronavirus infectious disease-2019 (COVID-19) pandemic continues to affect people worldwide. As of the end of July 2020, there are more than 14 million confirmed cases, with more than 0.6 million deaths (1). While the numbers of cases and deaths are expected to rise, a larger number of COVID-19 patients have recovered and have been discharged from medical facilities worldwide (1). However, little is known about post-COVID-19 sequelae among the discharged patients and related potential risk factors. Wuhan, China, was the first city to experience the emergence of COVID-19 (2). The central government launched timely public health strategies for virus control, including mandatory curfews and face coverings. At the medical facility level, COVID-19 testing was implemented and strict hospital discharge criteria were developed, including a mandatory 14-day period of post-hospital discharge clinical monitoring at regional shelter hospitals. M [a]any medical facilities continued to follow COVID-19 patients after primary hospital discharge, including the time periods during and after their stay at secondary shelter hospitals. We utilized these long-term follow-up data to investigate both the physical and psychological symptoms, including severe acute respiratory syndrome-related coronavirus 2 (SARS-CoV-2) immune recognition, among a large cohort of COVID-19 survivors released from four medical facilities in Wuhan.

## Methods

### COVID-19 Survivors Studied

This study investigated post-infection sequelae among all patients with confirmed COVID-19 infection who were discharged from four hospitals in Wuhan, China, between January 18 and March 29, 2020. These government-designated COVID-19 hospitals included Wuhan No.1 Hospital, Wuchang Hospital, Zhongshang Hospital, and Hubei Province Hospital. The hospitals were mandated to treat all infected patients regardless of disease severity (i.e., mild, severe, and critical). Standard hospital discharge criteria (3) included: (1) absence of fever for more than 3 days; (2) radiological evidence of significant resolution of pneumonia via CT scan; and (3) two sequential negative SARS-CoV-2 real-time quantitative PCR (RT-qPCR) tests on nasopharyngeal/oropharyngeal swab samples with at least a 24-h interval between sampling.

Early in the disease outbreak, little was known regarding the clinical characteristics, disease course, and mortality of COVID-19 infection, or the dynamics and range of viral transmission. Thus, all hospitalized patients who met the discharge criteria were immediately transferred to a Fangcang-like medical facility for a mandated 14-day period of clinical monitoring (4). The date of primary hospital discharge is the start of the follow-up. The last day of follow-up for the COVID-19 survivors included in this study was July 24, 2020. The data for this study are derived from case reports, medical records, and self-reports. This study was approved by the institutional ethics board of Wuhan No.1 Hospital, China (no. [2020] 6). Informed consent was obtained from each participant.

### Fangcang-Like Medical Facility and Its Discharge Criteria

Fangcang is a public health concept that was instituted for the first time in China during the SARS-CoV-2 outbreak of 2020. It was highly efficient at the mobilization of medical resources and dramatically reduced the burden on local medical capacity (4). Fangcang shelter hospitals are large-scale, temporary hospitals that are rapidly constructed by converting public venues (e.g., exhibition centers and football stadiums) into medical facilities equipped for basic medical care, frequent patient monitoring, and rapid patient assessment and referral. To maximize medical resources and to contain the rapidly emerging COVID-19 epidemic, government-designated hospitals in China discharged all COVID-19 patients directly to a Fangcang shelter hospital for a defined period of clinical observation, typically 14 days. This clinical observation serves as the initial phase of follow-up. After this observation period in the Fangcang hospital, patients were discharged home to self-quarantine if they met the following criteria: (a) no recurrence of any clinical symptom including respiratory-related inflammation and (b) negative SARS-CoV-2 RT-qPCR test on nasopharyngeal/oropharyngeal swab samples after 14 days.

COVID-19 survivors who developed any clinical symptom or sign during monitoring at the Fangcang shelter hospitals were immediately readmitted to hospitals. After discharge from Fangcang shelter hospitals, the community hospitals were responsible for clinical monitoring and diagnostic testings. When clinical symptoms occurred, or other medical circumstances required, or upon personal request, CT imaging and SARS-CoV-2 retesting were performed.

### SARS-CoV-2 Real-Time Quantitative PCR Test

RT-qPCR for SARS-CoV-2 was performed on nasopharyngeal/oropharyngeal swabs for viral detection. Viral RNA was extracted from the patients' nasopharyngeal/oropharyngeal swabs, sputum, and other samples, which were placed in a 56°C incubator for 30 min to inactivate the virus. Primer probes targeted three genes of SARS-CoV-2: open reading frame 1ab (*ORF1ab*) and genes of nucleocapsid proteins N and E (Table 1). The PCR buffer, reverse transcriptase enzyme, DNA polymerase, and gene primers were mixed together and added to a 96-well plate. The extracted RNA samples were then added to the wells, the plate sealed, and RT-qPCR amplification was performed as follows: one cycle at 40–45°C for 10 min, followed by 95°C for 3 min. Then, DNA denaturation and amplification proceeded for 45 cycles at 95°C for 15 s and 55–58°C for 30 s. The test results of SARS-CoV-2 were reported as positive or negative when the cycle threshold values remained below 44 or exceeded 43, respectively. The results in Table 1 were used to determine whether a sample was COVID-19-positive. This RT-qPCR test can detect SARS-CoV-2 nucleic acid standard substance within 400 copies/ml with a detection rate of 100%. Therefore, the sensitivity of the SARS-CoV-2 RT-qPCR is 400 copies/ml.

**Table 1:** Severe acute respiratory syndrome-related coronavirus 2 (SARS-CoV-2) real-time quantitative PCR (RT-qPCR) test results.

**ORF1ab**	**Gene N**	**Gene E**	**Result**
+	+	+	Positive
+	+	–	Positive
+	–	+	Positive
+	–	–	Positive only when a repeated test shows the same result
–	+	–	Suspicious. Recheck after a certain period
–	–	+	
–	–	–	Negative

For each patient, this SARS-CoV-2 RT-qPCR was performed twice on nasopharyngeal/oropharyngeal swab samples obtained with at least a 24-h interval between samples. Both tests must be negative in order to meet the discharge criteria of the hospital, as noted above. Subsequently, this test was performed at least once during Fangcang-medical monitoring; afterwards, this test was performed when clinical symptoms reoccurred, or upon personal requests, or for other reasons such as entering medical facilities and community centers.

### Colloidal Gold-Based Immunochromatographic Strip Assay

The colloidal gold-based immunochromatographic strip assay (IGCSA) was used for immunoglobulin G (IgG) and immunoglobulin M (IgM) detection. In brief, the IgM and IgG test cards were numbered sequentially. Anticoagulated (citrated) blood samples were centrifuged for 5 min at 500 × *g* and 10 μl of plasma was added to the sample well for 10–15 min. A test was considered positive when lines for the patient sample and the positive control sample appeared simultaneously. Samples in which a line developed only for the positive control sample were regarded as negative. Tests in which only the patient sample, and not the positive control sample, was positive were deemed invalid, requiring another test. Table 2 indicates the sensitivity and specificity of this assay for each immunoglobulin alone and in combination regarding SARS-CoV-2 detection in samples.

**Table 2:** Sensitivity and specificity of the colloidal gold-based immunochromatographic strip assay (ICGSA).

**Immunoglobulin test**	**Sensitivity (95% CI)**	**Specificity (95% CI)**
IgM	87.1% (83.9–91.3)	99.7% (98.4–100)
IgG	88.3% (85.6–92.0)	99.4% (98.0–99.8)
Combination of IgG and IgM	91.6% (88.6–93.4)	99.2% (97.6–99.7)

### Statistical Analysis

Continuous variables were expressed as median (interquartile range, IQR) and compared with the Mann–Whitney *U*-test; categorical variables were expressed as number (percentage) and compared by Fisher's exact test. *P* < 0.05 was regarded as statistically significant.

## Results

Between Jan 18 and Mar. 29, 2020, 3,677 hospitalized COVID-19 patients met the clinical discharge criteria and were discharged from the aforementioned four hospitals in Wuhan, China. All these COVID-19 survivors were included in our analyses. The median age was 59 years (IQR = 47–68, range = 10–98; 54.1% female). The survivors were followed for a median of 144 days (IQR = 135–157, range = 117–188; Table 3) by four independent medical teams. Among this cohort, 2,331 (63.4%) survivors had mild, 1,239 (33.7%) severe, and 95 (2.6%) had critical condition during their initial hospitalization. During this initial hospitalization, 3,570 (97.1%) survivors received antiviral therapy, 3,026 (82.3%) antibiotic treatment, 2,401 (65.3%) corticosteroids, 1,540 (41.9%) interferon nebulization treatment, and 1,445 (39.3%) γ-immunoglobulin treatment. Three thousand and sixty-six (83.4%) survivors were given a standard oxygen therapy via nasal catheter, 467 (12.7%) received high-flow nasal cannula therapy, 173 (4.7%) had non-invasive mechanic ventilation, and 30 (0.8%) required invasive mechanic ventilation. The median time from symptom onset to hospital admission was 8.0 days (IQR = 6.0–11.0). The median length of initial hospitalization was 17.0 days (IQR = 11.0–25.0).

**Table 3:** Clinical characteristics, retest positivity, and sequelae among discharged coronavirus infectious disease-2019 (COVID-19) patients.

**Characteristics**	**All patients (IQR/%)**	**Retested positive group (IQR/%)**	**Deceased group (IQR/%)**
No. of patients	3,677	45 (1.2)	37 (1.0)
Median age (years)	59.0 (47.0–68.0)	57 (50.0–64.0)	70.0 (56.0–79.0)
Gender			
Male	1,688 (45.9)	14 (31.1)	26 (70.3)
Female	1,989 (54.1)	31 (68.9)	11 (29.7)
Retest positivity	45 (1.2)	45 (100)	0
No. of new viral transmission	0	0	0
Severity (initial hospitalization)			
Mild	2,331 (63.4)	19 (42.2)	15 (40.5)
Severe	1,239 (33.7)	24 (53.3)	4 (10.8)
Critical	107 (2.9)	2 (4.5)	18 (48.6)
Treatment (initial hospitalization)			
Antiviral therapy	3,570 (97.1)	41 (91.1)	34 (91.9)
Antibiotic treatment	3,026 (82.3)	38 (84.4)	36 (97.3)
Corticosteroids	2,401 (65.3)	28 (62.2)	36 (97.3)
Interferon nebulization	1,540 (41.9)	15 (33.3)	5 (11.1)
γ-Immunoglobulin	1,445 (39.3)	1 (2.2)	3 (8.1)
Median time from symptom onset to admission (days, IQR)	8.0 (6.0–11.0)	8.0 (6.0–11.0)	10.0 (7.0–13.0)
Median time to hospitalization (days, IQR)	17.0 (11.0–25.0)	16.5 (11.0–24.0)	16.0 (10.0–23.0)
Median follow-up time (days, IQR)	144.0 (135.0–157.0)	150.0 (139.5–158.5)	33.0 (18.0–42.0)
**Sequelae**			
***Pulmonary function***	337 (9.2)		10 (27.0)
Shortness of breath	136	5	3
Cough/sputum	87	4	2
Pharyngitis/foreign body feeling	42	1	2
Dyspnea	30	7	3
Pulmonary fibrosis	21		
Lung damage	12		
Bronchitis	4		
COPD	3		
Hemoptysis	2		
***Cardiac function***	278 (7.6)		8 (21.6)
Chest pain/tightness	184	4	4
Palpitation	63	3	
Cardiac disease	14		2
Tachycardia	13	1	1
Angina pectoris	3		
Heart attack	1		1
***Neurologic function***	289 (7.9)		5 (13.5)
Insomnia	78	4	4
Joint pain/back pain/lumbago	71	3	
Fatigue	55		
headache/dizziness/poor memory	49		
Change of taste and smell	10		
Myalgia	8		
Impaired vision	5		
Leg numbness/finger stiffness	5		
Neuralgia	2		
Paralysis	2		
Tinnitus	2		
Confusion	1		1
Coma	1		
cerebral infarction	1		
***Endocrine system***	90 (2.4)		
Hair loss	67		
Bitter/dryness in mouth	12		
High blood sugar	6		
Diabetes	5		
***Gastrointestinal function***	42 (1.1)		1 (2.7)
Gastrointestinal complaints/poor appetite	31	3	1
Diarrhea	8		
Constipation	2		
Emesia	1		
***Dermatological system***	33 (0.9)		
Hidrosis	24		
Erythra	7		
Allergy	2		
***Hepatic system***	16 (0.4)		1 (2.7)
Hepatic insufficiency	8		1
Edema	7		
Antiadoncus	1		
***Kidney function***	12 (0.3)		1 (2.7)
Hypertension	6		
Kidney insufficiency	6		1
***Various***	66 (1.8)		5 (13.5)
Reduction of physical strength	64		5
Dryness/excessive secretion in eye	2		

During follow-up, 976 (26.5%) COVID-19 survivors had at least one sequelae (median age = 57, IQR = 47.8–56.4, range = 17–92; 59.0% female), the most common being chest pain/tightness (184, 5.0%), shortness of breath (136, 3.7%), and cough/sputum (87, 2.4%) (Table 3). Three hundred thirty-seven (9.2%) survivors had sequelae affecting pulmonary function, 278 (7.6%) had sequelae related to cardiac function, and 289 (7.9%) and 90 (2.4%) had sequelae impairing the neurologic system and endocrine function, respectively. Sequelae disposition by 10-year age interval of all the 3,677 survivors is included in Table 4. The incidence of post-COVID-19 sequelae of elderly COVID-19 survivors (age ≥60 years, *n* = 1,795) was slightly increased compared to that of young survivors (age <60, *n* = 1,882) [relative risk ratio (RR) = 1.05, 95% CI = 1.02–1.10, *p* = 0.007]. However, gender did not significantly influence the incidence of post-COVID-19 sequelae (Table 4). Additionally, 173 (4.7%) survivors self-reported diverse psychological symptoms such as anxiety (103, 2.8%), depression (70, 1.9%), and emotional instability (37, 1.0%). One hundred and thirty-two (3.6%) survivors refused to report personal feelings. Eight hundred two (21.8%) survivors were assessed by mental health care specialists and were deemed to have a clinically defined psychological condition. The psychological conditions of 136 (3.7%) survivors improved after psychological therapy.

**Table 4:** Sequelae disposition by 10-year age intervals of discharged coronavirus infectious disease-2019 (COVID-19) patients.

**Age intervals (years)**	**Sequelae Nr./Nr**.		**Relative risk ratio (95% CI, *p*-value)**
	**Male**	**Female**	**Male vs. female**
10–19	2/10	0/4	1.25 (0.92–1.70, *p* = 1)
20–29	7/56	14/91	0.98 (0.85–1.10, *p* = 0.81)
30–39	46/183	61/206	0.94 (0.83–1.06, *p* = 0.36)
40–49	77/256	78/250	0.98 (0.88–1.10, *p* = 0.84)
50–59	88/353	163/473	0.87 (0.80–0.95, *p* = 0.0036)
60–69	117/452	185/592	0.92 (0.86–1.00, *p* = 0.063)
70–79	48/265	56/256	0.95 (0.88–1.04, *p* = 0.323)
80–89	12/95	19/112	0.95 (0.85–1.06, *p* = 0.438)
>90	3/18	0/5	1.2 (0.98–1.48, *p* = 1)

**Figure 1:**
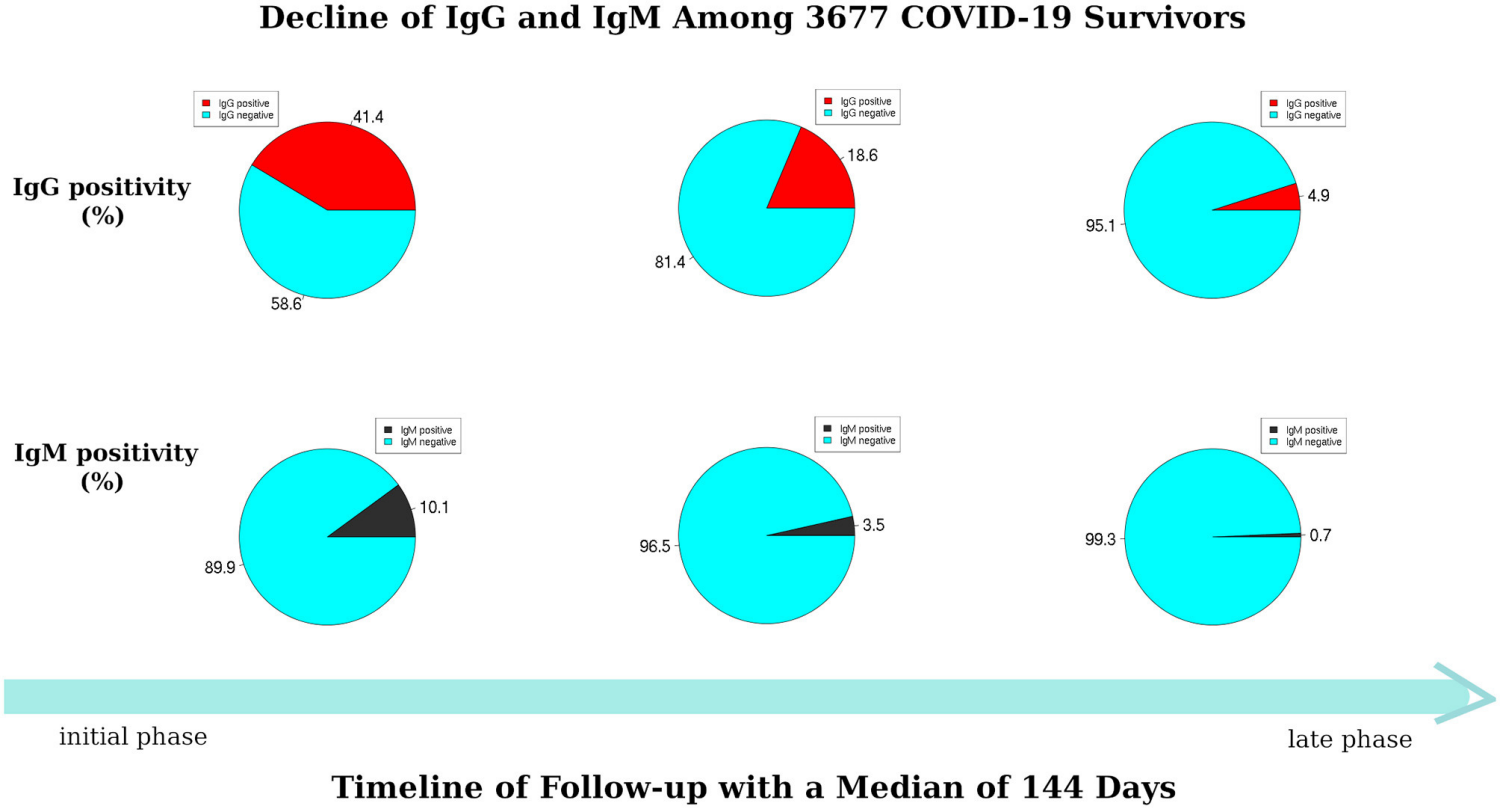
Dynamic decline of immunoglobulin G (IgG) and immunoglobulin M (IgM) among 3,677 coronavirus infectious disease-2019 (COVID-19) survivors during follow-up.

At the initial phase of the follow-up (days 1–45 post-hospital discharge), the results of the ICGSA for anti-SARS-CoV-2 viral immunoglobulins showed that 249 (6.8%) COVID-19 survivors were positive for both IgM and IgG, 1,274 (34.6%) were IgG-positive and IgM-negative, 121 (3.3%) were IgG-negative and IgM-positive, and 2,033 (55.3%) were negative for both IgG and IgM (Figure 1). At the late phase of the follow-up (days 100–150 post-hospital discharge), the IgG and IgM antibody positivity rates were reduced by 88.0% (95% CI = 84.2–90.4) and 93.2% (95% CI = 88.5–96.4), respectively. Specifically, only 25 (0.7%) survivors were positive for both IgG and IgM, 157 (4.3%) were IgG-positive and IgM-negative, none were IgG-negative and IgM-positive, and 3,495 (95.1%) were negative for both IgG and IgM (Figure 1).

During follow-up, 45 (1.2%) survivors retested positive for SARS-CoV-2 (median age = 57 years, IQR = 50–64, range = 25–81; 68.9% female; Table 3). None of these 45 was a health care worker, and none had taken medicine regularly after their initial hospital discharge. Of these, 25 survivors were immediately readmitted to hospitals and 20 remained at home under self-quarantine. Two of the 45 survivors had both IgG and IgM antibodies, 26 were IgG-positive and IgM-negative, two were IgG-negative and IgM-positive, and the remaining 15 were negative for both antibodies. The median duration between initial hospital discharge and retest positivity was 32.0 days (IQR = 28.0–40.0, range = 9–58; Table 3). Furthermore, 21 survivors in this retest-positive subgroup were asymptomatic, while 24 had at least one symptom associated with COVID-19, the most common being dyspnea, cough, and chest tightness. During their initial hospitalization, 19 of the 45 survivors had mild disease, 24 had severe condition, and two had critical condition. As of July 24, 2020, all 45 retest-positive survivors were alive. Twenty readmitted and retested positive survivors met the discharge criteria and were once again released to home quarantine. During follow-up, no new viral transmission was observed or reported.

During follow-up of the 3,677 COVID-19 survivors, 37 (1.0%) individuals died (median age = 70.0 years, IQR = 56.0–79.0, range = 31.0–98.0; 29.7% female; Table 3). None of the deceased was a health care worker. Thirty-one of the deaths were attributed to COVID-19, while six deaths were caused by comorbidities including diabetes, hepatobiliary tube cancer, heart attack, encephalorrhagia, epilepsy, and scurvy. The median duration from hospital discharge to death was 33.0 days (IQR = 18.0–42.0; Table 3). None of these deceased retested positive. Five of these 37 individuals had a worsened condition after hospital discharge and were therefore readmitted to the hospital; they died 4–15 days after readmission. The remaining 32 died at home. None of the deceased had taken any medicine regularly after initial hospital discharge and their psychological conditions had been stable. Except for one IgG-positive/IgM-negative individual, all other deceased individuals were IgG- and IgM-negative, indicating no immune system recognition of SARS-CoV-2. Within this deceased subgroup, 15 (40.5%) individuals had mild, four (10.8%) severe, and 18 (48.6%) had critical condition during their initial hospitalization. Also, during this initial hospitalization, 34 (91.9%) individuals were given antiviral treatments including arbidol, ganciclovir, oseltamivir, ribavirin, and hydroxychloroquine (Table 3), 36 (97.3%) individuals received antibiotics, and 36 (97.3%) were given corticosteroids.

## Discussion

To our knowledge, this study describes the longest duration of clinical follow-up in the largest cohort of discharged COVID-19 patients. Clearly, acute COVID-19 infection damages pulmonary function, but it has also been associated with the dysfunction of many other organ systems including the circulatory (5), cardiovascular (6), renal (7), gastrointestinal (8), endocrine (9), nervous (10), and skin (11) systems. In contrast, our study shows that diverse multi-organ functional impairments also occur well after hospital discharge in patients deemed recovered from primary acute infection. Specifically, during a mean follow-up time of 144 days, 976 (26.5%) of the COVID-19 survivors developed functional abnormalities of the cardiovascular, neurological, and endocrine systems. The risk of the development of such physical abnormalities was independent of age and gender. Furthermore, we show that 173 (4.7%) discharged patients had an associated psychological condition post-COVID-19 infection. These post-COVID-19 sequelae greatly impact the patients' long-term quality of life and will continue to strain the health care system.

Our study also reports a dramatic reduction of the anti-SARS-CoV-2 antibodies IgG and IgM during this long-term follow-up, which is consistent with the results of several recent studies (12–14). This result strongly suggests that adequate herd immunity may not develop or be maintained for a sufficient period to quell the pandemic. Such a finding supports the need to extend public health practices including social distancing procedures, face covering, and hygiene-based measures in order to limit viral transmission until an effective vaccine is developed and made widely available.

Our study describes a 1% overall mortality rate among Wuhan's COVID-19 survivors. Importantly, the majority of these deaths occurred in individuals who tested negative for both IgG and IgM. These results suggest that immune responses to SARS-CoV-19 infection may lower patients' risk of life-threatening post-discharge sequelae. The median age of this deceased group is 70 years, indicating that advanced age is not only a risk factor for death from primary COVID-19 infection but it also influences the mortality from post-COVID-19 sequelae. Fifteen of the 37 deceased persons had a mild course of COVID-19 during the initial hospitalization, indicating that the disease severity of primary COVID-19 infection is not the sole factor contributing to mortality in the post-COVID-19 period. Furthermore, the majority of deaths during follow-up were those of male survivors (26, 70.3%), implying that female gender could play an important role in survival during the post-COVID-19 period.

Lastly, we report a 1.2% rate of COVID-19 retest positivity among all COVID-19 survivors, with no new viral transmission. We cannot confirm whether or not retest-positive people are able to infect others; however, as long as the appropriate social health measures are practiced, reinfection via retest-positive people can likely be avoided.

Our study has some limitations. Being a national multicenter study, the findings must be further verified by international studies. Secondly, this study was not designed or able to determine the effects of treatment on the follow-up outcome. Thirdly, a recent study revealed the clinical characteristics of family members for COVID-19 infection (15). However, our study was not able to determine the post-COVID-19 sequelae-related genetic relationship. This warrants further investigation.

In summary, our results indicate that persistent and often severe morbidity is prevalent among COVID-19 survivors. Individuals with such post-viral sequelae may have reduced quality of life, including lost productivity, and may continue to strain health care systems. Closer follow-up of COVID-19 survivors with prompt medical intervention for developing sequelae may improve the long-term outlook for these individuals.

## Data Availability Statement

The raw data supporting the conclusions of this article will be made available by the authors, without undue reservation.

## Ethics Statement

The studies involving human participants were reviewed and approved by the institutional ethics board of Wuhan No.1 Hospital, China (No. [2020] 6). Informed consent was obtained from each participant. The patients/participants provided their written informed consent to participate in this study.

## Author Contributions

QM, FW, YY, GH, JL, CK, QZ, and SG contributed to the acquisition, analysis, or interpretation of data. QM, YY, JL, and AB drafted the manuscript. QM and JL did the statistical analysis. AB, FW, GH, LW, XY, and LZ contributed to the critical revision of the manuscript for important intellectual content. FW obtained funding. YY and GH gave administrative, technical, or material support. LW, XY, and JL helped with conception design and supervision. All authors contributed to the article and approved the submitted version.

## Conflict of Interest

The authors declare that the research was conducted in the absence of any commercial or financial relationships that could be construed as a potential conflict of interest.
